# Contributions to the retrospective (1987-1993) and prospective (2008-2013) epidemiological characterization of HIV-AIDS cohort in Constanța county

**DOI:** 10.1186/1471-2334-14-S7-P21

**Published:** 2014-10-15

**Authors:** Iulia Gabriela Șerban, Sorin Rugină

**Affiliations:** 1 Regional HIV/AIDS Center Constanța, Romania; 2 Faculty of Medicine, “Ovidius” University, Constanța, Romania

## Background

The chosen theme is relevant to show the progress in addressing public health services currently in Romania, especially the care of HIV-AIDS patients in the HIV-AIDS Regional Centre from Constanța.

## Methods

We aimed to organize the existing case law in a database, which represents a prerequisite for further steps to elucidate the epidemiological process. We established three groups of patients: 1. Registered in the 1987-1993 period, which is the main cohort of children infected through nosocomial transmission – included in Study I, retrospective. 2. Registered in the 2008-2012 period – included in Study II, prospective. 3. Registered in the January – 30 June 2013 period – included in Study III, prospective.

We implemented a model of active epidemiological surveillance, with correlation of all factors, including new and old cases, based on monthly data reporting frequency. We processed the existing database, the records of consultations; we evaluated health surveys, laboratory data, clinical observation sheets multi-tracking, the register of psychological evaluation.

## Results

During 1987 to 1990 the population was uninformed on the HIV-AIDS phenomenon; they later became familiar with the existence of infection, transmission patterns and healthcare opportunities (after 1990). The route of transmission was: transfusion, injections (parenteral, 84.549%). The treatments were performed in the pediatric ward, and the parenteral treatments in other sections (dystrophic, premature baby, swings). Orphan patients came from the wards dystrophic and premature, mostly being institutionalized or abandoned children. The epidemic was first rural and subsequently urban epidemic. Analyzing the pyramid of the ages, first time pediatric population prevails, subsequent adult population. The determinant virus is "wild”, new in circulation, belonging to subtype F1 overwhelmingly (99.9%). The diagnosis of the cases evolved from clinical diagnosis and HIV testing by ELISA, by modern means of today (phenotype, genotyping, imaging, resistance tests). The mortality rate fell spectacularly, especially due to the introduction of antiretroviral therapy. Constanța is the second regional center HIV-AIDS in the country. Since 2009 a "dedicated" pathological department is functioning.

## Conclusion

The originality of the study: a descriptive presentation from the registers and studied observation sheets was transformed into a database, which was presented in the study. Constanța has come to represent a model for surveillance and monitoring of HIV nationally and internationally, many of the models of care, programs, were expanding to other counties and countries.

